# Independent association of SOT-composite with berg balance scale scores in Chinese stroke patients: a retrospective cross-sectional study

**DOI:** 10.3389/fneur.2026.1918753

**Published:** 2026-10-08

**Authors:** Wenhua Zheng, Wei Shen, Mengyun Li, Gengmao Ma, Minhan Xie, Qingying Leng, Ruixian Zhang, Churong Liu, Xiaolin Zeng, Hanbo Chen

**Affiliations:** 1 Institute for Brain Research and Rehabilitation, China Normal University, Guangzhou, China; 2 Department of Rehabilitation Therapy, Guangdong 999 Brain Hospital, Guangzhou, China; 3 Department of Rehabilitation Medicine, Guangdong China Energy Construction Electric Power Hospital, Guangzhou, China; 4 Department of Acupuncture and Tuina, Gushengtang Wushan General Outpatient Department, Guangzhou, China

**Keywords:** balance, berg, rehabilitation, SOT-composite score, stroke

## Abstract

**Objective:**

Stroke-related balance dysfunction affects 61–83% of patients, yet the quantitative relationship between sensory integration capacity and functional balance remains inadequately characterized. This study investigated the association between Sensory Organization Test (SOT) composite scores and Berg Balance Scale (BBS) performance in Chinese stroke patients.

**Methods:**

This retrospective cross-sectional study analyzed data from 339 stroke patients who received treatment at Guangdong 999 Brain Hospital between January 2021 and December 2023. Exposure variable was SOT-Composite score (0–100 points, measured via NeuroCom Smart EquiTest within 48 h). Outcome variable was Berg Balance Scale total score (0–56 points, assessed within 48 h). Two models were constructed: a Core model adjusting for age, gender, diagnosis, and disease duration, and a Sensitivity model additionally adjusting for Brunnstrom lower-extremity stage and weight symmetry index. Tertile stratification of SOT-Composite scores was performed descriptively to explore dose–response patterns.

**Results:**

In the Sensitivity model, each 1-point increase in SOT-Composite score was associated with a 0.2745-point higher Berg Balance Scale score (95% CI: 0.171–0.386, *p* < 0.001; Core model: *β* = 0.2471, 95% CI 0.137–0.357, *p* < 0.001). Standardized coefficient analysis in the Sensitivity model showed Brunnstrom lower-extremity stage had the strongest association (standardized *β* = 0.4562, *p* < 0.001), followed by SOT-Composite score (standardized *β* = 0.2527, *p* < 0.001). Among sensory subsystems, visual, somatosensory, and vestibular processing ratios were all significantly associated with balance performance (unstandardized *β* = 0.18, 0.16, and 0.15 BBS points per unit ratio change, respectively).

**Conclusion:**

SOT-Composite score was independently associated with Berg Balance Scale performance in stroke patients, even after adjustment for multiple covariates. Whether targeted sensory training can improve balance outcomes warrants evaluation in future prospective intervention studies.

## Introduction

Stroke remains a leading cause of death and disability worldwide, with continuously rising incidence rates. According to the Global Burden of Disease Study 2019 (GBD 2019), there were 12.2 million new stroke cases globally, with over 101 million prevalent cases (1). In China, the stroke burden is particularly severe. The China Stroke Surveillance Report 2021 indicated that the age-standardized incidence rate of stroke was 246.8 per 100,000 person-years, with a mortality rate of 114.8 per 100,000 person-years (2). Balance dysfunction represents one of the most common functional impairments following stroke, with reported occurrence rates ranging from 61 to 83% (3). The Berg Balance Scale (BBS) serves as the gold standard tool for assessing functional balance capacity and plays a crucial role in stroke rehabilitn evaluation (4–6).

The Sensory Organization Test (SOT) composite score is a critical indicator for evaluating the human sensory system’s integration capacity. It quantifies the relative contributions of vestibular, visual, and somatosensory systems in maintaining balance to assess an individual’s postural control abilities (7–9). SOT systematically manipulates visual and support surface information, forcing subjects to rely on different sensory inputs to maintain balance, thereby identifying sensory integration deficits. Research has demonstrated significant clinical value of SOT composite scores in neurodegenerative diseases such as Parkinson’s disease and Alzheimer’s disease (10, 11). In Parkinson’s disease patients, SOT composite scores effectively distinguish fallers from non-fallers, while in patients with cognitive impairment, this indicator correlates with subcortical structural atrophy (11, 12). However, there is currently insufficient evidence regarding the application of SOT composite scores in stroke populations (13).

Existing research on balance function assessment in stroke patients predominantly focuses on single sensory modalities or traditional balance tests, with relatively limited large-sample studies examining the relationship between multisensory integration capacity and functional balance performance (14). Although studies have explored the effects of cognitive tasks on sensory organization (15) and the relationship between balance function and fall risk (16), these investigations present methodological controversies, particularly regarding confounding factor control and sample size selection (17, 18). Furthermore, the considerable variation in adjustment variables across different studies affects result comparability and generalizability (19).

Based on the current evidence gaps, this cross-sectional study enrolled 339 stroke patients from China between January 2021 and December 2023 to systematically evaluate the association between SOT composite scores and Berg Balance Scale total scores. While previous studies (e.g., Oliveira et al., Fiedorová et al.) have examined the SOT-BBS relationship in smaller samples (*n* = 21–42), this study extends the evidence base through: (1) to our knowledge, the largest sample size to date (*n* = 339) examining this relationship specifically in a Chinese stroke population, with substantially greater statistical power; (2) implementation of multi-level adjustment models with more comprehensive covariate control than previously reported; (3) quantification of the differential contributions of individual sensory subsystems (somatosensory, visual, and vestibular) to balance function; and (4) exploratory item-level correlation analysis suggesting that sensory integration capacity may preferentially relate to complex balance tasks requiring precise postural control. The findings will provide novel theoretical foundations for balance function assessment in stroke patients, offer important references for developing individualized rehabilitation training programs, and contribute new clinical evidence for understanding sensory-motor integration mechanisms following stroke.

## Methods

### Study population

This retrospective cross-sectional study analyzed data from 339 stroke patients who received treatment at Guangdong 999 Brain Hospital between January 2021 and December 2023. All participants were from China and stroke diagnosis was confirmed by head CT or MRI imaging, including acute cerebral infarction and intracerebral hemorrhage. Inclusion criteria were: (1) age ≥18 years; (2) first-ever stroke; (3) admission within 3 months of onset; (4) ability to cooperate with balance function assessment. Exclusion criteria were: (1) previous stroke history; (2) severe cognitive impairment preventing cooperation with assessments; (3) severe cardiopulmonary disease (e.g., heart failure or chronic obstructive pulmonary disease) precluding safe completion of standardized balance testing; (4) comorbid neurological diseases; (5) missing data for exposure variable (SOT-Composite score) or outcome variable (Berg Balance Scale total score). All clinical data were obtained from the hospital electronic medical record system and collected by trained research personnel using standardized data extraction forms to ensure data completeness and accuracy. Of the eligible patients, 339 had complete data for the primary exposure (SOT-Composite score) and outcome variable (Berg Balance Scale total score) as well as all covariates in the Core model (age, gender, disease duration, stroke type, and affected side), yielding the complete-case sample size of 339 for primary models. Three secondary variables not used in the Core or Sensitivity models had variable-specific missing proportions: standing balance (10.32%), Holden Functional Ambulation Category (8.26%), and sitting balance (0.88%). Little’s MCAR test (*χ*^2^ = 184.53, df = 38, *p* < 0.001) indicated that missingness in secondary variables deviated from completely random; see Supplementary Table S1 for full participant flow and missingness accounting by variable.

### Sample size calculation

All patients admitted to Guangdong 999 Brain Hospital between January 2021 and December 2023 who met the inclusion and exclusion criteria were included; no sampling was performed. A *post hoc* power analysis, based on a previous pilot study showing a correlation coefficient of 0.3 between SOT-composite and Berg Balance Scale scores with *α* = 0.05 and power = 80%, indicated that a minimum of 84 participants would be required to achieve 80% statistical power. Our achieved sample of 339 participants substantially exceeded this *post hoc* statistical power requirement.

### Variables

The exposure variable, SOT-Composite score, was measured using the NeuroCom Smart EquiTest system (NeuroCom International, Inc., Clackamas, OR, United States) within 48 h of admission. The Sensory Organization Test (SOT) systematically evaluates the contribution of visual, vestibular, and somatosensory systems to balance control through six specific testing conditions: Condition 1 (eyes open, fixed surface and visual surround)—testing baseline postural stability; Condition 2 (eyes closed, fixed surface)—evaluating somatosensory dependence by eliminating visual input; Condition 3 (eyes open, fixed surface, sway-referenced visual surround)—assessing the ability to suppress inaccurate visual information; Condition 4 (eyes open, sway-referenced surface, fixed visual surround)—testing the ability to use visual information when somatosensory input is inaccurate; Condition 5 (eyes closed, sway-referenced surface)—measuring vestibular function with absent visual and diminished somatosensory inputs; Condition 6 (eyes open, sway-referenced surface and visual surround)—evaluating vestibular function with conflicting visual and somatosensory cues.

For each condition, patients were tested for three 20-s trials while standing barefoot on the force platform with arms at sides. The system measured postural sway by recording center of gravity (COG) displacements and calculated an equilibrium score (0–100 points) for each trial, where higher scores indicate less postural sway and better balance control. The SOT-Composite score (ranging from 0–100 points) was calculated using a weighted average of all conditions, with particular emphasis on the more challenging conditions (4–6). Additionally, three sensory analysis ratios were calculated: somatosensory ratio (condition 2/condition 1), visual ratio (condition 4/condition 1), and vestibular ratio (condition 5/condition 1), representing the relative contribution of each sensory system to overall balance function. Higher SOT-Composite scores indicate better sensory integration capacity. SOT-Composite was recorded as a continuous variable in the database and additionally categorized into tertiles (low, middle, high) to explore dose–response relationships.

The outcome variable, Berg Balance Scale total score, was assessed by certified physiotherapists within 48 h of admission. The scale comprises 14 standardized balance tasks with a total score ranging from 0–56 points, where higher scores indicate better balance function. Assessment was conducted non-blinded but independently from exposure variable measurement. Both assessments were completed within the first 48 h after admission, in accordance with standard unit protocol; however, our retrospective database did not systematically record the exact time-of-day or minute-level interval between the two assessments, nor did it consistently document the sequence of administration for each patient. Based on unit workflow during the study period, BBS assessment generally preceded SOT testing, although exceptions may have occurred and cannot be fully verified retrospectively. Likewise, although no rehabilitation session was scheduled between the two assessments per standard protocol, we cannot completely exclude the possibility of brief bedside mobility activity in isolated cases, as this was not systematically logged. This assessment-timing heterogeneity, compounded by variable post-onset admission timing (within 3 months), is discussed as a limitation below.

Covariates were selected based on previous literature and clinical relevance: age and gender as basic demographic characteristics; disease duration reflecting acute phase status; diagnosis type (cerebral infarction vs. intracerebral hemorrhage) and affected side influencing functional recovery patterns; Brunnstrom lower limb stage (I-VI) assessing motor function recovery level; MMSE total score reflecting cognitive function status; weight symmetry index evaluating postural control capacity. Missing data proportions for individual Core-model covariates were zero (primary exposure and outcome) or negligible (<1% for demographic/clinical variables). Secondary variables not included in the primary analyses had higher missing proportions: standing balance (10.32%), Holden Functional Ambulation Category (8.26%), and sitting balance (0.88%). See Supplementary Table S1 for complete variable-specific missingness details and Little’s MCAR test result.

### Ethic statement

This retrospective study was approved by the Ethics Committee of Guangdong 999 Brain Hospital (Ethics Approval No. 2021–01-040) for the analysis of anonymized electronic medical record data collected during standard clinical care between January 2021 and December 2023. The research process strictly adhered to the ethical principles of the Declaration of Helsinki. Given the retrospective nature of the study using anonymized clinical data without additional interventions or patient contact, informed consent was waived by the institutional ethics committee. All patient data were de-identified prior to analysis to ensure adequate privacy protection. Data collection, storage, and analysis complied with relevant legal and regulatory requirements and were used solely for the scientific purposes of this study.

### Statistical analysis

The prespecified primary analysis was the association between SOT-Composite score and Berg Balance Scale total score in the Core and Sensitivity models. All sensory-ratio analyses, item-level correlations, and tertile categorizations were exploratory and hypothesis-generating; they were not adjusted for multiplicity beyond the Benjamini-Hochberg procedure applied to the 14 item-level correlations. Continuous variables were presented as mean ± standard deviation, while categorical variables were expressed as frequency (percentage). Baseline characteristics were compared across SOT-Composite score tertiles using analysis of variance for continuous variables and chi-square test for categorical variables. The association between SOT-Composite score and Berg Balance Scale total score was analyzed using a two-model approach based on a conceptual distinction between confounders and possible mediators. The Core model adjusted for age, sex, diagnosis, and disease duration—variables with no plausible mediating role between SOT-Composite and balance function. The Sensitivity model additionally included Brunnstrom lower-extremity stage and weight symmetry, explicitly identified as possible mediators rather than conventional confounders, given their potential to lie on the causal pathway between sensory integration and balance performance. Throughout this manuscript, the term ‘independent association’ refers strictly to association conditional on the measured covariates in each model, not a causal claim. The robustness of results was evaluated by comparing effect estimates across different adjustment strategies. Sensitivity analysis was performed by converting SOT-Composite score into tertile categories and conducting linear regression analysis to verify the consistency with continuous variable results and confirm the dose–response relationship between SOT-Composite score and Berg total score. All statistical analyses were performed using R software (version 4.3.0). Linear regression assumptions were verified through residual analysis, including normality of residuals (Shapiro–Wilk test), homoscedasticity (Breusch-Pagan test), and absence of multicollinearity (variance inflation factor <5). Sensitivity analyses addressing the bounded nature of the Berg Balance Scale (0–56 range) were conducted: HC3 heteroscedasticity-consistent robust standard errors and bootstrap confidence intervals (1,000 replications) were computed for the Sensitivity-model SOT-Composite coefficient. Model diagnostics and detailed results are provided in Supplementary Table S2. Two-sided *p*-values less than 0.05 were considered statistically significant. All point estimates are reported with 95% confidence intervals and exact *p*-values; no secondary significance threshold was employed.

A detailed accounting of participant enrollment, exclusion by reason, variable-specific missing data, and Little’s MCAR test result is provided in Supplementary Table S1.

Item-level associations between SOT-Composite score and the 14 individual BBS item sums were assessed using Pearson correlation. *p*-values were adjusted for the 14 comparisons using the Benjamini-Hochberg false discovery rate (FDR) procedure, with adjusted *α* = 0.05/14 ≈ 0.0036 for individual tests.

## Results

A total of 339 stroke patients were stratified into three equal tertile groups (*n* = 113 each) based on SOT-Composite scores. Significant differences were observed across groups: patients in the high tertile were younger (52.06 ± 12.53 vs. 57.77 ± 12.36 years, *p* = 0.003), had a higher proportion of males (80.9% vs. 67.6%, *p* = 0.044), and demonstrated superior cognitive function (MMSE: 22.94 ± 6.30 vs. 20.58 ± 6.09, *p* = 0.005); weight symmetry improved progressively across tertiles, with the high group achieving near-normal levels (101.73 ± 22.71% vs. 87.83 ± 42.30%, *p* < 0.001); Berg Balance Scale scores were significantly higher in the high tertile (36.97 ± 17.40 vs. 29.98 ± 15.03, *p* = 0.002); the proportion of bilateral hemiplegia decreased from low to high tertiles (27.0% vs. 11.3%, *p* = 0.044). Disease duration (*p* = 0.453), stroke type distribution (*p* = 0.990), and Brunnstrom lower limb staging (*p* = 0.493) showed no significant differences between groups, though motor recovery demonstrated a favorable trend in higher SOT-Composite tertiles (Table 1).

**Table 1:** Baseline characteristics of study participants stratified by SOT-Composite score tertiles.

Variables	SOT-composite score tertile			*p*-value
	Low (*n* = 113)	Middle (*n* = 113)	High (*n* = 113)	
Continuous variables				
Age (years)	57.77 ± 12.36	54.69 ± 12.34	52.06 ± 12.53	0.003
Disease duration (days)	15.19 ± 15.50	15.70 ± 16.94	13.26 ± 13.70	0.453
MMSE total score	20.58 ± 6.09	22.72 ± 5.37	22.94 ± 6.30	0.005
Weight symmetry	87.83 ± 42.30	100.47 ± 22.30	101.73 ± 22.71	<0.001
SOT-composite score	58.96 ± 19.51	73.64 ± 2.35	83.51 ± 3.19	<0.001
Berg balance scale total score	29.98 ± 15.03	35.89 ± 14.26	36.97 ± 17.40	0.002
Categorical variables, *n* (%)				
Gender				0.044
Male	76 (67.6)	89 (78.8)	91 (80.9)	
Female	37 (32.4)	24 (21.2)	22 (19.1)	
Stroke type				0.990
Ischemic stroke	88 (77.5)	88 (77.9)	88 (78.3)	
Hemorrhagic stroke	25 (22.5)	25 (22.1)	25 (21.7)	
Affected side				0.044
Left	48 (42.3)	51 (45.1)	52 (46.1)	
Right	35 (30.6)	41 (36.3)	48 (42.6)	
Bilateral	30 (27.0)	21 (18.6)	13 (11.3)	
Brunnstrom stage (lower limb)				0.493
Stage V	57 (50.5)	46 (40.7)	38 (33.9)	
Stage VI	25 (22.5)	28 (24.8)	33 (29.6)	
Stage IV	17 (15.3)	26 (23.0)	24 (21.7)	
Stage III	11 (9.9)	12 (10.6)	14 (12.2)	
Stage II	2 (1.8)	1 (0.9)	2 (1.7)	
Stage I	0 (0.0)	0 (0.0)	1 (0.9)	

Continuous variables are presented as mean ± standard deviation. Categorical variables are presented as frequency (percentage). *p*-values were calculated using one-way ANOVA for continuous variables and chi-square test for categorical variables. SOT, sensory organization test; MMSE, Mini-Mental State Examination.

Multivariate linear regression analysis demonstrated a significant positive association between SOT-Composite score and Berg Balance Scale total score in both the Core model (*β* = 0.2471, 95% CI 0.137–0.357, *p* < 0.001, adjusted *R*^2^ = 0.063) and the Sensitivity model (*β* = 0.2745, 95% CI 0.171–0.386, *p* < 0.001, adjusted *R*^2^ = 0.187) (Table 2). When analyzed as a continuous variable, each 1-point increase in SOT-Composite score was associated with a 0.2745-point higher Berg Balance Scale score (95% CI: 0.171–0.386, *p* < 0.001) in the sensitivity model. Further analysis of sensory subsystems revealed differential contributions to balance function: the somatosensory ratio showed a significant association with Berg Balance Scale scores (*β* = 0.16, 95% CI: 0.06–0.26, *p* = 0.001), as did the visual ratio (*β* = 0.18, 95% CI: 0.08–0.28, *p* < 0.001) and vestibular ratio (*β* = 0.15, 95% CI: 0.05–0.25, *p* = 0.003) in the fully adjusted model (Table 3). These findings suggest that all three sensory systems were significantly associated with balance function. Although the unstandardized coefficient for the visual ratio was numerically slightly higher, the 95% confidence intervals overlapped substantially, and no formal statistical comparison between coefficients was performed; given that these are unstandardized coefficients on differing measurement scales, this numerical difference should not be interpreted as evidence of a significantly stronger contribution from visual processing. Because these three ratios share Condition 1 as a common denominator and are therefore collinear, they were entered in three separate regression models (each adjusted for the same Core covariates) rather than in a single combined model; consequently, no combined-model VIF is reported for these ratios. Tertile analysis was consistent with a dose–response relationship: compared to the low tertile, the middle tertile showed initial significance (5.91 points, *p* = 0.005) that attenuated and lost significance after full adjustment (3.29 points, *p* = 0.08), while the high tertile maintained consistent significance across all models, with a statistically significant 4.81-point higher BBS score in the high tertile compared with the low tertile (95% CI: 1.01–8.61, *p* = 0.01). This represents a cross-sectional between-group difference of approximately 8.6% of the total BBS score range and should not be interpreted as a within-patient treatment effect or directly equated with the minimal clinically important difference (MCID), which is derived from longitudinal, pre-post intervention data in different populations. The tertile-based comparison shown here represents a descriptive cross-sectional stratified analysis; it is not a formal sensitivity analysis addressing the bounded nature of the BBS outcome (0–56 range). The robustness of the primary continuous-variable estimate was evaluated separately through HC3 robust and bootstrap inference, reported above and in Supplementary Table S2.

**Table 2:** Association between SOT-composite score and berg balance scale (BBS) total score.

Model	Adjustment set	Unstandardized *β*	95% CI	*p* value	Standardized *β*	Adjusted *R*^2^
Core model	Age, sex, diagnosis, disease duration	0.2471	0.137 to 0.357	<0.001	0.2363	0.063
Sensitivity model	Core covariates + Brunnstrom lower-limb stage + weight-bearing symmetry	0.2745	0.171 to 0.386	<0.001	0.2527	0.187

BBS = Berg Balance Scale; SOT = Sensory Organization Test; CI = confidence interval. Data are based on complete-case analysis (*n* = 339); no missing data were present for SOT-Composite, BBS total score, age, sex, diagnosis, disease duration, Brunnstrom lower-limb stage, or weight-bearing symmetry. The core model adjusts for age, sex, diagnosis, and disease duration. The sensitivity model additionally adjusts for Brunnstrom lower-limb stage and weight-bearing symmetry to evaluate the robustness of the SOT-Composite–BBS association after accounting for motor recovery stage and postural loading asymmetry. Unstandardized *β* represents the estimated change in BBS total score per 1-point increase in SOT-Composite score. Diagnostic statistics (residual normality, heteroscedasticity, and, for the sensitivity model, influence measures) and robustness checks for the SOT-Composite coefficient in the sensitivity model (HC3 robust 95% CI; bootstrap 95% CI, 1000 resamples) are provided in Supplementary Table S2.

**Table 3:** Multiple regression analysis of sensory ratios and berg balance scale scores in stroke patients.

Sensory ratio	Unadjusted *β* (95% CI)	*p*-value	Adjusted I *β* (95% CI)	*P*-value	Adjusted II *β* (95% CI)	*P*-value
Somatosensory ratio	0.20 (0.10, 0.31)	<0.001***	0.20 (0.09, 0.30)	<0.001***	0.16 (0.06, 0.26)	0.001***
Visual ratio	0.20 (0.09, 0.30)	<0.001***	0.20 (0.09, 0.31)	<0.001***	0.18 (0.08, 0.28)	<0.001***
Vestibular ratio	0.17 (0.06, 0.27)	0.002**	0.17 (0.06, 0.28)	0.003**	0.15 (0.05, 0.25)	0.003**

Adjust I model is adjusted for sex, height, occupation, age, handedness, education, and disease duration (days).

Adjust II model is adjusted for age, sex, disease duration (days), diagnosis, hemiplegia side, Brunnstrom stage (lower limb), MMSE total score, and weight-bearing symmetry (backward).

*β*: Unstandardized regression coefficient (BBS points per unit change in ratio). 95% CI: 95% confidence interval.

**p < 0.01, ***p < 0.001.

MMSE, mini-mental state examination; CI, confidence interval.

Comparative analysis of standardized coefficients in the fully adjusted model (Table 4) revealed that Brunnstrom lower limb stage (standardized *β* = 0.38, *p* < 0.001) showed a stronger association with Berg Balance Scale scores than SOT-Composite score (standardized *β* = 0.25, *p* < 0.001), followed by MMSE total score (standardized *β* = 0.14, *p* = 0.007), disease duration (standardized *β* = 0.13, *p* = 0.009), and age (standardized *β* = −0.12, *p* = 0.037). This suggests that while sensory integration capacity is significantly associated with balance function, motor recovery status shows the strongest association with functional balance in stroke patients (Table 4).

**Table 4:** Standardized regression coefficients for factors associated with berg balance scale scores in stroke patients.

Variable	*β*	95% CI	*P*-value
Brunnstrom stage (lower limb)	−0.38	(−0.48, −0.28)	<0.001***
SOT-Composite score	0.25	(0.13, 0.36)	<0.001***
MMSE total score	0.14	(0.04, 0.25)	0.007**
Disease duration (days)	0.13	(0.03, 0.23)	0.009**
Age	−0.12	(−0.22, −0.01)	0.037*
Affected side	−0.10	(−0.20, 0.01)	0.068
Stroke type	−0.07	(−0.18, 0.03)	0.167
Weight symmetry (backward)	−0.07	(−0.18, 0.04)	0.240
Gender	0.04	(−0.05, 0.14)	0.386

Variables are ordered by absolute magnitude of standardized regression coefficients (*β*). All variables were simultaneously entered into a multiple regression model with Berg Balance Scale total score as the dependent variable. 95% CI = 95% confidence interval.

*p < 0.05, **p < 0.01, ***p < 0.001.

MMSE, mini-mental state examination; SOT, sensory organization test.

Further exploratory item-level analysis showed that SOT-Composite scores were most strongly correlated with Berg items requiring complex balance control: standing with feet together (*r* = 0.290, FDR-adjusted *p* < 0.001), standing with one foot in front (*r* = 0.248, FDR-adjusted *p* < 0.001), and placing alternate foot on step (*r* = 0.234, FDR-adjusted *p* < 0.001). In contrast, lower correlations were observed with items assessing basic functional movements: sitting unsupported (*r* = 0.039, FDR-adjusted *p* = 0.474), standing to sitting (*r* = 0.103, FDR-adjusted *p* = 0.064), and standing unsupported (*r* = 0.120, FDR-adjusted *p* = 0.032). Given the 14 item-level comparisons performed, *p*-values were adjusted using the Benjamini-Hochberg false discovery rate (FDR) procedure. This exploratory, hypothesis-generating pattern suggests that SOT-Composite score may be more strongly associated with challenging balance tasks than with basic mobility functions (Table 5).

**Table 5:** Pearson correlations between SOT-composite score and individual berg balance scale items.

Berg balance scale item	Pearson r	Raw *P*-value	FDR-adjusted P (BH)
Standing with feet together	0.290	5.7 × 10^−8^	8.0 × 10^−7^***
Standing with one foot in front (tandem stance)	0.248	3.7 × 10^−6^	2.6 × 10^−5^***
Placing alternate foot on step or stool (stepping)	0.234	1.4 × 10^−5^	5.9 × 10^−5^***
Turning 360 degrees	0.231	1.8 × 10^−5^	5.9 × 10^−5^***
Standing with eyes closed	0.229	2.1 × 10^−5^	5.9 × 10^−5^***
Reaching forward with outstretched arm	0.219	4.6 × 10^−5^	1.1 × 10^−4^***
Turning to look behind (left & right)	0.214	7.1 × 10^−5^	1.4 × 10^−4^***
Standing on one foot (single-leg stance)	0.211	9.1 × 10^−5^	1.6 × 10^−4^***
Retrieving object from floor	0.199	2.3 × 10^−4^	3.6 × 10^−4^***
Transfers	0.188	5.1 × 10^−4^	7.1 × 10^−4^***
Sitting to standing (item)	0.181	8.1 × 10^−4^	1.0 × 10^−3^**
Standing unsupported	0.120	0.027	0.032 *
Standing to sitting	0.103	0.059	0.064
Sitting unsupported	0.039	0.474	0.474

Items are ordered by the magnitude of correlation coefficients. *p*-values were adjusted for multiple comparisons across 14 items using the Benjamini–Hochberg false discovery rate (FDR) procedure. Significance stars are based on FDR-adjusted *p*-values. *p < 0.05, **p < 0.01, ***p < 0.001. SOT, Sensory Organization Test.

Residual diagnostics on the Core model indicated departure from normality (Shapiro–Wilk *p* < 0.001) and heteroscedasticity (Breusch-Pagan *p* = 0.0476). The Sensitivity model showed departure from normality (W = 0.9283, *p* = 1.08 × 10^−11^) but homoscedasticity (Breusch-Pagan *χ*^2^ = 7.1960, *p* = 0.4088). Maximum Cook’s distance (0.0601) and leverage value (0.0955) indicated no unduly influential individual observations. Given the heteroscedasticity and non-normality, we computed HC3 robust standard errors and 95% CIs (bootstrap, 1,000 replications) for the Sensitivity-model SOT-Composite coefficient: HC3 95% CI = 0.1674–0.3816 (compared to OLS 95% CI = 0.171–0.386), and bootstrap 95% CI = 0.1711–0.3864. These closely matched the parametric OLS estimate, indicating robust inference. Complete diagnostics are presented in Supplementary Table S2.

## Discussion

In this large-scale cross-sectional analysis of 339 stroke patients utilizing multiple linear regression models, we investigated the association between sensory integration capacity (assessed by SOT-Composite scores, the exposure) and balance function (measured by Berg Balance Scale scores, the outcome). The substantial sample size ensured adequate statistical power and robust findings. Our analysis revealed that each 1-point increase in SOT-Composite score was associated with a 0.2745-point higher Berg Balance Scale score in the sensitivity model (95% CI: 0.171–0.386, *p* < 0.001). Patients in the high SOT-Composite tertile showed a 4.81-point higher adjusted Berg Balance Scale score compared with those in the low tertile (95% CI: 1.01–8.61, *p* = 0.01). This cross-sectional between-group difference should not be interpreted as a within-patient treatment effect or equated with the minimal clinically important difference. These findings are broadly consistent with Matsumoto et al.’s 2024 study, which established that Berg Balance Scale cutoff values of 46.5–50.5 points were associated with fall risk in community-dwelling stroke patients (20), however, that cohort differed from ours in care setting (community-dwelling versus acute inpatient) and timing of assessment, which may limit direct comparability. Our findings are also consistent with Fiedorová et al.’s work demonstrating an SOT cutoff value of 60 points (AUC 0.686) and Berg Balance Scale cutoff values of 35–42 points associated with fall risk in stroke patients (21), noting that population characteristics and assessment timing also differed between studies. Furthermore, our dose–response relationship findings resonate with Chen et al.’s 2024 scoping review emphasizing the importance of balance training in preventing falls in stroke patients (22), and are supported by Jiang et al.’s 2024 meta-analysis confirming that balance training significantly enhances balance and walking function in stroke patients (23).

Although our study identified a significant linear relationship between SOT-Composite scores and Berg Balance Scale performance, it is important to note that these two assessments evaluate fundamentally different aspects of balance function and cannot substitute for each other. The SOT primarily measures sensory integration capacity under controlled laboratory conditions, focusing on the physiological mechanisms of balance control, while the Berg Balance Scale assesses functional balance performance in practical tasks relevant to daily activities. The Sensitivity-model adjusted *R*^2^ was 0.187, reflecting the variance in BBS total score explained jointly by SOT-Composite and all included covariates—not variance shared between SOT and BBS specifically. A partial *R*^2^ isolating the contribution of SOT-Composite alone was not calculated in this analysis.

Regarding the ethical considerations of performing both tests on stroke patients, we carefully designed our assessment protocol to minimize patient burden. The SOT testing required approximately 15–20 min, while the Berg Balance Scale assessment took 15–25 min, with adequate rest periods provided between tests. Both assessments were part of the standard clinical evaluation at our center, conducted for therapeutic decision-making rather than solely for research purposes. The combined information from both tests allows for more comprehensive and personalized rehabilitation planning. Per the department’s standard assessment protocol documented in nursing and physiotherapy records, all patients received detailed explanations about the purpose and procedures of both tests and were monitored for signs of fatigue or discomfort throughout the assessments. No adverse events related to these evaluations were documented in the medical record during the study period.

Our findings are highly consistent with the seminal study by Oliveira et al. (2011), which compared Berg Balance Scale and Sensory Organization Test scores in 21 patients with hemiparesis after first-ever ischemic stroke versus 21 healthy controls, demonstrating significant impairments in both sensory integration and balance control in stroke patients (24). Although Oliveira et al. employed a case–control design with a smaller sample size (*n* = 42), while our study utilized a larger cross-sectional design (*n* = 339), both studies consistently confirmed the significant association between SOT and Berg Balance Scale scores. Our study’s advantages include greater statistical power and more comprehensive covariate adjustment strategies. However, methodological differences exist with Fiedorová et al. (21) ROC analysis study, which focused on clinical validity verification of SOT, Berg Balance Scale, and Falls Efficacy Scale using sensitivity and specificity analyses to determine optimal cut-off values for predicting fall risk. This difference may stem from distinct research objectives: we focused on quantifying the linear relationship between sensory integration and balance function, while Fiedorová et al. aimed to determine optimal cut-off values for predicting fall risk. From a neuroscience mechanism perspective, balance control deficits caused by sensory integration disorders primarily involve abnormal reweighting processes of vestibular, visual, and proprioceptive information, a mechanism particularly prominent within the first year post-stroke, as revealed by the Oliveira study (24). The monotonic association observed is consistent with a potential role of sensory integration in balance function; however, it does not by itself provide intervention guidance and should be interpreted as hypothesis-generating.

The monotonic, approximately linear association observed in this study is consistent with a dose–response pattern, but no threshold or breakpoint effect was formally tested for clinical assessment and intervention strategies in stroke rehabilitation. Compared to existing literature that predominantly focuses on qualitative descriptions of sensory integration disorders (25). Our study quantifies the linear association strength between SOT scores and balance function in a Chinese stroke cohort. Our findings raise the hypothesis that SOT-Composite scores could potentially inform future patient stratification for individualized rehabilitation strategies; however, this stratification approach, including any specific cutoff values, has not been tested in this study and would require validation through prospective intervention trials before informing clinical resource allocation or rehabilitation guideline recommendations. Future research should prioritize validating the effectiveness of individualized intervention protocols based on SOT scores through multicenter prospective cohort studies, exploring sensory integration recovery patterns in different stroke subtypes, and investigating the application potential of emerging technologies such as virtual reality in sensory integration training (26) to advance precision rehabilitation medicine.

The clinical value of this study lies in its quantification of the relative contributions of different sensory subsystems to balance function in stroke patients. Multivariate regression analyses revealed that SOT-Composite score (*β* = 0.25, *p* < 0.001) was significantly associated with balance performance, while Brunnstrom stage (*β* = 0.38, *p* < 0.001) showed a stronger association. This pattern suggests that both motor recovery and sensory integration may be relevant correlates of balance function; whether rehabilitation protocols combining both domains would improve outcomes remains to be tested in future intervention studies. Our findings of differential sensory contributions—with somatosensory (*β* = 0.16), visual (*β* = 0.18), and vestibular (*β* = 0.15) ratios all independently associated with balance function—provide a novel framework for targeted sensory rehabilitation. Particularly valuable is our item-level correlation analysis showing that sensory integration capacity more strongly influences complex balance tasks (standing with feet together: *r* = 0.290; tandem stance: *r* = 0.248) than basic mobility functions (sitting unsupported: *r* = 0.039; standing to sitting: *r* = 0.103). This pattern is exploratory and suggests that foundational and advanced balance tasks may have differential associations with sensory integration capacity. Whether a stratified rehabilitation approach emphasizing sensory training for advanced balance tasks would improve outcomes remains an open question that requires prospective intervention trials to address; this study cannot evaluate such causal claims. Incorporating standardized sensory integration evaluation into stroke rehabilitation protocols is a direction worth exploring, but any recommendations regarding resource allocation efficiency or protocol/guideline modification would require validation through prospective intervention trials before informing clinical or policy decisions.

This study demonstrates significant advantages in research design and data analysis strategies, establishing a solid methodological foundation for investigating the relationship between sensory integration and balance function. First, the large sample size of 339 stroke patients ensures adequate statistical power, substantially exceeding the sample sizes of previous similar studies (typically 60–100 participants) (22, 24) and enhancing both the reliability and generalizability of our findings. Second, we employed multiple linear regression models with systematic adjustment for important confounding factors including stroke type, disease duration, and age, effectively controlling the impact of potential bias on association estimates.

Third, the comprehensive analysis of three sensory subsystem ratios (somatosensory, visual, and vestibular) and item-level BBS correlations provides exploratory, hypothesis-generating information beyond aggregate SOT-Composite scores. Addressing methodological deficiencies of inadequate confounding adjustment identified in previous research (27). Additionally, we conducted tertile-based sensitivity analyses to explore the dose–response relationship, providing scientific evidence for individualized intervention strategies (28, 29). These methodological features enhance the reliability of our findings within the scope of a single-center, retrospective, cross-sectional design; they do not establish new methodological standards for the field, and replication in independent, prospective, multicenter cohorts is needed.

Because the exact interval, sequence, and intervening clinical activity between SOT and BBS assessments were not systematically logged in our retrospective database, we cannot fully exclude the possibility that some assessments were separated by longer intervals, administered in reversed order, or accompanied by brief interim mobility activity, any of which could introduce measurement noise into the estimated SOT-BBS association. Future prospective studies employing standardized, time-stamped assessment protocols are needed to address this limitation.

Because inclusion required the ability to cooperate with and complete SOT testing, patients with the most severe motor, cognitive, visual, or balance impairment were likely underrepresented, potentially limiting generalizability and possibly affecting the estimated strength of the SOT-BBS association. As this was a retrospective study, complete records of patients excluded for inability to complete SOT testing were not systematically retained, precluding a precise quantification of this potential selection effect.

As a cross-sectional observational study, we can only detect associations between sensory integration and balance function rather than establish causality. Despite the significant clinical value of this study, several limitations warrant acknowledgment. First, our inclusion and exclusion criteria limit the generalizability of our findings: excluding patients with severe cognitive impairment may underestimate the potential role of sensory integration training in stroke patients with dementia, a common exclusion criterion in stroke rehabilitation research that affects representativeness of results (30), excluding those with severe cardiopulmonary diseases (consistent with the exclusion criteria specified in Methods) prevents direct application of our findings to stroke populations with comorbid heart failure or chronic obstructive pulmonary disease, while excluding patients with other neurological disorders limits the extrapolation of results to stroke patients with concurrent Parkinson’s disease or multiple sclerosis. Second, this single-center study has limited generalizability, requiring validation through additional similar studies, a limitation widely recognized in stroke rehabilitation research (31). Furthermore, our study population consisted primarily of Chinese individuals, necessitating caution when applying these findings to other ethnic groups, as different races may exhibit physiological differences in sensory integration capacity and balance control strategies, with existing research demonstrating that racial differences may be associated with stroke recovery outcomes (32). As a cross-sectional observational study, we can only detect associations between sensory integration and balance function rather than establish causality, an inherent limitation of observational study designs (33, 34). Finally, although we adjusted for several important confounding factors, our retrospective dataset did not include several clinically relevant indicators of stroke severity and balance-related impairment—including NIHSS score, lesion location, hemispatial neglect, lower-extremity muscle strength, assistive-device use, and rehabilitation dose/exposure—raising the possibility of residual confounding. In addition, because Brunnstrom stage and weight symmetry index may partly mediate rather than purely confound the SOT-BBS association, our adjusted estimates should be interpreted with caution regarding possible over adjustment.

## Data Availability

The original contributions presented in the study are included in the article/Supplementary material, further inquiries can be directed to the corresponding author.
