# Supplementary material for: Independent association of SOT-composite with berg balance scale scores in Chinese stroke patients: a retrospective cross-sectional study
Source: Front Neurol. 2026 Oct 8;17:1918753. doi: 10.3389/fneur.2026.1918753 (PMC13686881; doi:10.3389/fneur.2026.1918753)
Supplement: Supplementary file 1 [file Table_1.docx]

**Supplementary Table S1: Participant Source and Data Completeness**

This retrospective cross-sectional study was based on the electronic clinical database of the Department of Rehabilitation Therapy, Guangdong 999 Brain Hospital, and included patients assessed between January 2021 and December 2023. The dataset available for analysis contained 339 eligible inpatients with stroke, representing the complete set of eligible records available in the extracted database. Because the database extraction provided to the investigators did not retain the full source screening log from the original hospital record query process, the total pre-screening denominator and the exclusion counts at each screening stage could not be reconstructed retrospectively.

**Data completeness**

The primary exposure (SOT-Composite), the primary outcome (Berg Balance Scale total score), and all core covariates in the main adjusted model (age, sex, diagnosis, disease duration) had no missing values. Missingness in the analytic dataset was limited to three secondary clinical variables:

| Variable | Missing (n/N) | % Missing |
| --- | --- | --- |
| Standing balance | 35/339 | 10.32% |
| Holden Functional Ambulation Classification | 28/339 | 8.26% |
| Sitting balance | 3/339 | 0.88% |

**Missing-data mechanism**

Little's MCAR test was performed to formally evaluate the missingness pattern: χ² = 184.53, df = 38, P < 0.001. This result indicates that the missingness pattern was not fully consistent with missing completely at random (MCAR). Interpretation of analyses involving these secondary variables should account for this limitation. The primary exposure–outcome association reported in this study (SOT-Composite–BBS) is unaffected, as none of the variables involved in the primary and sensitivity models had missing data.

Abbreviations: MCAR = missing completely at random.
