# Supplementary material for: Independent association of SOT-composite with berg balance scale scores in Chinese stroke patients: a retrospective cross-sectional study
Source: Front Neurol. 2026 Oct 8;17:1918753. doi: 10.3389/fneur.2026.1918753 (PMC13686881; doi:10.3389/fneur.2026.1918753)
Supplement: Supplementary file 2 [file Table_2.docx]

**Supplementary Table S2. Full Model Diagnostics**

Core model

BBS_total ~ SOT_composite + age + sex + diagnosis + disease duration (N = 339)

| Diagnostic | Value |
| --- | --- |
| Shapiro–Wilk normality test | W = 0.9205, P = 1.99 × 10⁻¹² |
| Breusch–Pagan heteroscedasticity test | χ² = 11.1995, P = 0.0476 |

Sensitivity model

Core model + Brunnstrom lower extremity stage + weight symmetry (N = 339)

| Diagnostic | Value |
| --- | --- |
| Shapiro–Wilk normality test | W = 0.9283, P = 1.08 × 10⁻¹¹ |
| Breusch–Pagan heteroscedasticity test | χ² = 7.1960, P = 0.4088 |
| Maximum Cook's distance | 0.0601 |
| Maximum leverage | 0.0955 |
| HC3 robust 95% CI (SOT-Composite) | 0.1674 to 0.3816 |
| Bootstrap 95% CI (SOT-Composite, 1000 resamples) | 0.1711 to 0.3864 |

Footnote: Both models showed statistically significant deviation from normality in residuals (Shapiro–Wilk P < 0.001), which is common in moderate-to-large clinical samples and does not invalidate ordinary least squares estimation under the Gauss–Markov framework given the sample size (N = 339). The Breusch–Pagan test was non-significant in the sensitivity model (P = 0.4088), indicating no strong evidence of heteroscedasticity for the final adjusted model. HC3 robust standard errors and bootstrap resampling were used as sensitivity checks; both produced confidence intervals highly consistent with the model-based estimate, supporting the stability of the SOT-Composite coefficient. No observation exceeded conventional influence thresholds (Cook's D > 1; leverage thresholds based on 2(k+1)/n), indicating the association was not driven by a small number of influential cases.

Abbreviations: SOT = Sensory Organization Test; BBS = Berg Balance Scale; CI = confidence interval.
